# Targeting pancreatic and ovarian carcinomas using the auristatin-based anti-CD70 antibody–drug conjugate SGN-75

**DOI:** 10.1038/sj.bjc.6605816

**Published:** 2010-07-27

**Authors:** M C Ryan, H Kostner, K A Gordon, S Duniho, M K Sutherland, C Yu, K M Kim, A Nesterova, M Anderson, J A McEarchern, C-L Law, L M Smith

**Affiliations:** 1 Seattle Genetics, Inc. 21823 – 30th Drive SE, Bothell, WA 98021, USA

**Keywords:** CD70, pancreatic, ovarian, antibody–drug conjugate

## Abstract

**Background:**

CD70 is an ideal target for antibody-based therapies because of its aberrant high expression in renal carcinomas and non-Hodgkin lymphomas and its highly restricted expression in normal tissues. The expression profiling of CD70 in carcinomas has been limited because of the lack of a CD70-specific reagent that works in formalin-fixed paraffin-embedded (FFPE) tissues.

**Methods:**

We generated murine monoclonal antibodies (mAbs) specific for CD70 and validated their specificity by western blot analysis and developed a protocol for immunohistochemistry on FFPE tissues. CD70+ tumour cell lines were used for testing the anti-tumour activity of the anti-CD70 antibody–drug conjugate, SGN-75.

**Results:**

We report novel detection of CD70 expression in multiple cancers including pancreatic (25%), larynx/pharynx (22%), melanoma (16%), ovarian (15%), lung (10%), and colon (9%). Our results show that pancreatic and ovarian tumour cell lines, which express high levels of endogenous or transfected CD70, are sensitive to the anti-tumour activity of SGN-75 *in vitro* and *in vivo*.

**Conclusion:**

Development of murine mAbs for robust and extensive screening of FFPE samples coupled with the detection of anti-tumour activity in novel indications provide rationale for expanding the application of SGN-75 for the treatment of multiple CD70 expressing cancers.

Pancreatic cancer is the fourth leading cause of cancer death among men and women in the United States. In 2009, it was estimated that 42 470 individuals in the US will be diagnosed with pancreatic cancer and over 35 240 will die from the disease (Jemal *et al*, 2009). The 5-year relative survival rate for pancreatic cancer is only 5.1% and it has changed little in the last three decades. For ovarian cancer, 21 550 women will be diagnosed in 2009 and 14 600 women will die of the disease (Jemal *et al*, 2009). The overall 5-year relative survival rate was 45.5% for 1996–2004. Worldwide, around 140 000 women die of ovarian cancer every year (National Cancer Institute SEER Cancer Statistics). Both ovarian and pancreatic cancer patients have great unmet need for better therapeutic options.

CD70 (*TNFSF7*), a member of the tumour necrosis factor (TNF) superfamily, is a type II integral membrane protein and the ligand for CD27 (Goodwin *et al*, 1993; Bowman *et al*, 1994; Hintzen *et al*, 1994b). CD70 is transiently expressed in antigen-activated T and B lymphocytes (Hintzen *et al*, 1994a, 1995; Lens *et al*, 1996) and its interaction with CD27 regulates T- and B-cell functions (Jacquot *et al*, 1997; Agematsu *et al*, 1998; Hendriks *et al*, 2000; Arens *et al*, 2001; Tesselaar *et al*, 2003; Xiao *et al*, 2004). CD70 protein expression is very limited in non-lymphoid organs and has been reported only in stromal cells of the thymic medulla (Hishima *et al*, 2000) and mature dendritic cells (Bullock and Yagita, 2005). Aberrant expression of CD70 in solid tumours and haematologic malignancies has also been described by various studies (Agathanggelou *et al*, 1995; Lens *et al*, 1999; Diegmann *et al*, 2005; Law *et al*, 2006). CD70 has been reported to be highly expressed in renal cell (clear and papillary types) (Law *et al*, 2006), thymic (Hishima *et al*, 2000), nasopharyngeal (Agathanggelou *et al*, 1995), and brain (astrocytoma, glioblastoma) tumours (Wischhusen *et al*, 2002). It is also widely expressed in Hodgkin and non-Hodgkin lymphomas (HL and NHL), Waldenstrom's macroglobulinemia, chronic lymphocytic leukaemia, and multiple myeloma (MM) (Al Saati *et al*, 1989; Ranheim *et al*, 1995; Treon *et al*, 2007; McEarchern *et al*, 2008).

We have previously shown targeting CD70 in renal cell carcinomas (clear cell and papillary types) and in haematologic malignancies (HL, MM) using anti-CD70-drug conjugates (Law *et al*, 2006; Oflazoglu *et al*, 2008; Alley *et al*, 2009). Both chimeric and humanised anti-CD70 monoclonal antibody (mAb) 1F6 conjugated to a microtubule-disrupting auristatin have been shown to be active in these tumours. With the limited normal tissue distribution, aberrant expression in tumours, and cell surface expression with no observed shedding of the antigen, CD70 is an attractive target for antibody–drug conjugate (ADC)-based therapy. The ADCs generated against CD30 (brentuximab vedotin (SGN-35)) and erbB2 (Herceptin-DM1) are currently in clinical trials with tumour reductions and minimal toxicity observed (Senter, 2009). The clinical potential of empowered antibodies has heightened the pursuit of ADCs for additional targets and tumour indications. SGN-75, an auristatin-based anti-CD70 ADC, recently entered a phase I clinical trial for patients with renal cell carcinoma and NHL. In this study, we expanded our analysis of CD70 expression in various types of carcinomas to identify new potential indications for SGN-75.

## Materials and methods

### Reagents and cell lines

Peroxidase-conjugated and Cy5-conjugated goat anti-mouse IgG were purchased from Jackson ImmunoResearch Laboratories (West Grove, PA, USA). 3,3′,5,5′-Tetramethylbenzidine was purchased from Pierce (Rockford, IL, USA). Basal media, penicillin, streptomycin, FBS and sodium hypoxanthine, aminopterin and thymidine (HAT) were purchased from Invitrogen (Carlsbad, CA, USA). Cloning factor and complete mini-protease inhibitors were from Roche Diagnostics (Indianapolis, IN, USA), and fetal clone I was from Hyclone (Logan, UT, USA). Pancreatic (Capan-1, Capan-2, SU.86.86, Panc-1, HPAF-II, MiaPaCa-2), ovarian (OVCAR-3, SK-OV-3, CaOV3, TOV-21G), endometrial (AN3CA), renal (786-O), and colon (NCI-H716) carcinoma cell lines were obtained from American Tissue Culture Collection (ATCC, Manassas, VA, USA). All cell lines were grown in culture at 37°C using recommended cell culture media according to ATCC.

### Recombinant CD70 purification

Chinese hamster ovary DG44 cells were transfected with a construct encoding the cynomolgus CD70 (cyno CD70) extracellular domain (ECD) with a FLAG tag and the soluble recombinant protein was harvested from the media. The cynoCD70 ECD was purified by affinity and size-exclusion chromatography using an ÄKTA explorer FPLC (GE Healthcare, Piscataway, NJ, USA). Briefly, the media were concentrated and treated with 0.05% (v/v) Tween-20, 0.25% Triton X-100 (Sigma, St Louis, MO, USA). Samples were loaded on to M2 anti-FLAG affinity column (GE Healthcare). FLAG cynoCD70 was then eluted from the column with 0.1 M glycine, pH 3.5 and then dialysed against PBS, pH7.4. The pooled protein was then loaded on to Superdex 200 (GE Healthcare) pre-equilibrated with PBS, pH 7.4 to remove low molecular weight contaminants.

### Antibody generation

Female BALB/c mice were immunised subcutaneously with denatured recombinant CD70 ECD using TiterMax adjuvant (Sigma). B cells were harvested from immunised spleens and fused to P3-X63.Ag8 myeloma cells using a standard PEG fusion protocol (Goding, 1996). Hybridomas were cultured in 80% IMDM with 4 mmol l^−1^ L-glutamine, 10% fetal clone I, 10% cloning factor supplemented with penicillin, streptomycin and 1 × HAT. Hybridoma culture supernatants were tested for binding to purified recombinant CD70 protein by ELISA and for binding to formalin-fixed CD70-positive cells using a FMAT8200 instrument (Applied Biosystems, Foster City, CA, USA). Positive hybridomas were then screened on formalin-fixed paraffin-embedded (FFPE) cell pellets to identify hybridomas that react with denatured CD70 for immunohistochemistry (IHC) analysis. The top two hybridomas, designated SG-21.1C1 and SG-21.5D12, were cloned in semi-solid media using a ClonePixFL instrument (Genetix, New Milton, Hampshire, UK) and then expanded for purification. Antibodies were affinity purified using Mabselect Sure resin (Amersham, Piscataway, NJ, USA), eluted using 25 mM acetate (pH=3.4) and dialysed in PBS.

### ELISA and cell-based binding assays

Immunosorb 96-well plates were coated with 2 *μ*g ml^−1^ of recombinant cynoCD70, washed with PBS+1% Tween (PBS-T), and blocked with PBS-T plus 1% (w/v) BSA. CD70-coated plates were incubated with hybridoma culture supernatants for 2 h at room temperature (RT), washed 5 × with PBS-T, and incubated with peroxidase conjugated goat-anti-mouse IgG. After incubation with secondary antibody, plates were washed, incubated with 3,3′,5,5′-tetramethylbenzidine substrate, and stopped with an equal volume of 1 mol l^−1^ H_2_S0_4_. For cell-based binding assays, HEK 293:pcDNA and HEK 293:cynoCD70-transfected cell lines were plated at a density of 20 000 cells per well in black 96-well plates (Applied Biosystems). Hybridoma culture supernatants were added to wells at 1/10th volume and supplemented with 100 ng ml^−1^ of Cy5-conjugated goat-anti-mouse IgG. Test wells that differentially bound to HEK 293:cynoCD70 but not HEK 293:pcDNA were ranked using a FMAT8200 instrument (Applied Biosystems).

### Membrane preparation and western blot analysis

Harvested cells were resuspended in hypotonic lysis buffer (10 mM Tris, pH 7.4, 10 mM KCl, 1.5 mM MgCl_2_) with 1 mM DTT plus protease inhibitors and cell membranes were disrupted using a dounce homogeniser. Nuclei were pelleted by centrifugation at 10 000 × **g** for 30 min. The supernatant was collected and membrane fractions were pelleted by centrifugation at 100 000 × **g** for 30 min and then solubilised in NP40 lysis buffer (0.5% NP40, 50 mM Tris, pH 8.0, 150 mM NaCl, 5 mM EDTA) containing protease inhibitors. Purified FLAG-CD70 ECD and membrane preps of HEK 293F, HEK 293F/cynoCD70, and 786-O cells were run on a 4–20% gradient Tris-Glycine gel (Life Technologies, Carlsbad, CA, USA) under reducing conditions and transferred to PVDF membrane. For western blot analysis, membranes were first blocked in PBS-T+1% BSA and then incubated with 0.5 *μ*g ml^−1^ of purified SG-21.1C1 or SG-21.5D12 in PBS-T+1% BSA at RT for 1 h. A rabbit polyclonal antibody against *β*-actin (Cell Signaling, Danvers, MA, USA) was used as protein loading control. The blots were washed, incubated with HRP-conjugated anti-mouse IgG and then washed and developed using the West Pico ECL reagent (Pierce/Thermo Fisher Scientific, Rockford, IL, USA).

### Immunohistochemistry

Tumour microarrays (TMAs) and normal tissue microarrays were obtained from a commercial source (USBiomax Inc., Rockville, MD, USA). Formalin-fixed cells spun down into pellets and paraffin-embedded, FFPE tissue samples or TMAs sectioned on glass slides were deparaffinised and rehydrated. EDTA-based antigen retrieval was carried out at 95–99°C for 40 min before incubation with the primary antibodies (1 *μ*g ml^−1^ for 30–45 min at 25°C). Isotype-matched murine antibodies (Sigma) were used as negative controls for background staining. For manual staining, an anti-murine secondary antibody conjugated to HRP was used with detection using diaminobenzidine (DAB) as chromogen. For automated staining, either Refine DAB Kit or Alkaline Phosphatase-Red was used with the BondmaX autostainer (Leica Microsystems, Bannockburn, IL, USA) for detection of primary antibody binding. Tissue microarray slides were analysed and scored by a pathologist and images were taken using Zeiss Axiovert 200 M microscope (Carl Zeiss, Inc., Thornwood, NY, USA).

### Flow cytometric analysis

Cells were incubated for 30 min on ice with PE-conjugated murine anti-CD70 antibodies (BD Pharmingen, San Diego, CA, USA), washed with cold staining medium, and evaluated with a Becton Dickinson FACScan flow cytometer. Quantification of CD70 copy number on the cell surfaces was determined using a murine anti-CD70 mAb (Ki-24, BD Pharmingen) and the DAKO QiFiKit flow cytometric indirect assay as described by the manufacturer (DAKO A/S, Glostrup, Denmark).

### Detection of human CD70 expression by PCR

The expression of human CD70 in pancreatic carcinoma was examined by PCR with cDNA preparations obtained from tumour tissues. Primers that spanned the coding regions (exons 1 to 3) for *TNFSF7* and to amplify the housekeeping gene, *GAPDH*, were synthesised by Operon Biotechnology (Huntsville, AL, USA).

Human *GAPDH* – 5′-CCACCCATTGGCAAATTCCATGGCA-3′ (forward) and 5′-TCTAGACGGCAGGTCAGGTCCACC-3′ (reverse).

Human *TNFSF7* – 5′-GCTGGTCCCCTGACAGGTTGAA-3′ (forward) and 5′-CCTTCTCTTGTCCTGCCACCAC-3′ (reverse).

PCR was carried out in an Eppendorf Mastercycler (Westbury, NY, USA) with 1 *μ*l cDNA and 200 nM primers. The samples were heated for 5 min at 94°C and then one group was used to amplify CD70 (94°C for 45 s, 62°C for 45 s, and 72°C for 45 s for 35 cycles followed by an incubation at 72°C for 5 min to yield a product of 852 bp), whereas the other was used for GAPDH (94°C for 45 s, 69°C for 45 s, and 72°C for 45 s for 23 cycles followed by an incubation at 72°C for 5 min to yield a product of 580 bp). PCR products were visualised by electrophoresis on a 1% agarose gel. The identities of the PCR products were confirmed by sequencing.

### Cytotoxicity assay

Tumour cells were incubated with h1F6 (anti-CD70 mAb) and h1F6-mcMMAF drug conjugate (SGN-75) for 96 h at 37°C. A non-binding ADC (cAC10-mcMMAF) was used as a negative control. Cell viability was measured by CelltiterGlo (Promega Corporation, Madison, WI, USA) according to the manufacturer's instructions. Cells were incubated for 25 min at room temperature with the CellTiter-Glo reagents and luminescence was measured on a Fusion HT fluorescent plate reader (Perkin Elmer, Waltham, MA, USA). Results are reported as IC_50_, the concentration of compound needed to yield a 50% reduction in viability compared with vehicle-treated cells (control=100%).

### Generation of stable pancreatic cell line expressing CD70

A full-length cynomolgus gene for *TNFSF7*, previously isolated for non-human primate binding studies, was used to create stable HEK 293F and MiaPaCa-2 transfectants for antibody screening and *in vivo* studies, respectively. The cynomolgus *TNFSF7* gene is >90% homologous to human *TNFSF7* (based on sequencing) and SGN-75 binds with the same affinity to cyno CD70 and human CD70 (unpublished data). The pancreatic cell line MiaPaCa-2 was transfected with 1 *μ*g of linearised pcDNA3.1-cynoCD70 plasmid using Lipofectamine 2000 reagent (Life Technologies) in six-well tissue culture plates according to the manufacturer's protocol. Transfection media was replaced with selective media containing 800 *μ*g ml^−1^ of Geneticin (Life Technologies) 24 h after transfection. Media was replaced every 2–3 days until the outgrowth of positive transfectants. The cell surface expression of CD70 was then assessed by flow cytometry. CD70-positive bulk cultures were cloned by limiting dilution cloning and grown for at least 4 weeks in growth media without selective antibiotic to identify CD70-positive stable clones.

### *In vivo* activity study

Nude (*nu/nu*) female mice (seven animals per group) were implanted with MiaPaCa-2/CD70 tumour pieces by trocar into the right lateral flank. Dosing with either SGN-75 or non-binding control ADC (3 mg kg^−1^) started when tumours reached 100 mm^3^ (four intraperitoneal injections at 4-day interval). Tumour volumes were monitored using calipers and animals were euthanised when tumour volume reached ∼800 mm^3^. Median tumour volume plots were continued for each group until one or more animals were killed. Kaplan–Meier curves were generated to reflect survival of each animal per group and statistical analysis was carried out using Log-rank (Mantel–Cox) test (GraphPad Prism, GraphPad Software, San Diego, CA, UAS). All animal procedures were carried out under a protocol approved by the Institutional Animal Care and Use Committee in a facility accredited by the Association for Assessment and Accreditation of Laboratory Animal Care.

## Results

### Validation of anti-CD70 mAb as an IHC reagent

Profiling of CD70 across a broad array of tumour samples has been limited by the lack of an available antibody that reacts specifically with CD70 in FFPE tissues. Our testing of commercially available anti-CD70 mAbs as well as rabbit polyclonal antibodies generated against various CD70 peptides showed either non-specific binding or no binding at all in FFPE tissues (data not shown). Therefore, we embarked on a *de novo* antibody campaign to develop a high-quality IHC reagent for detection of CD70 in normal and pathologic tissue samples. Screening of a panel of anti-CD70 hybridomas by IHC using FFPE CD70+ and CD70− cell pellets identified two hybridomas that showed selective binding to CD70+ cells. These hybridomas, designated 1C1 and 5D12, were then cloned and purified for further characterisation.

**Figure 1:**
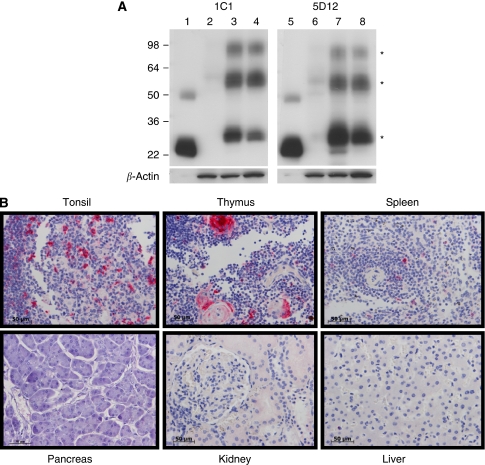
Characterisation of anti-CD70 1C1 and 5D12. (**A**) Western blots containing FLAG-tagged CD70 ECD (lanes 1 and 5) and membrane extracts from HEK 293 cells (lanes 2 and 6), HEK 293:CD70 transfectants (lanes 3 and 7) and 786-O cells (lanes 4 and 8) were reacted with mAb 1C1 (left panel) or mAb 5D12 (right panel). The mAb 1C1 and 5D12 specifically bound recombinant CD70 (lane 1 and 4) and three distinct bands (^*^) from CD70+ membrane extracts (lanes 3, 4, 7, and 8). Extracts from parental HEK 293 cells were negative as expected (lane 2 and 6). The *β*-actin control shows comparable quantities of protein were loaded for each membrane extract (lower panels). (**B**) IHC analysis on FFPE normal human tissues showing limited expression of CD70 in lymphoid tissues. Red stain indicates presence of CD70+ cells. 1C1 mAb was used for these set of images. × 400 magnification, scale bar=50 *μ*m.

The specific binding of mAbs 1C1 and 5D12 to CD70 was analysed by western blot analysis using purified FLAG-tagged CD70 ECD and membrane-enriched samples from HEK 293F parental, CD70-transfected HEK 293F cells and a CD70+ renal cell carcinoma cell line (786-O). Figure 1A shows that both mAbs bound to the purified CD70 ECD (lanes 1 and 5) and to three distinct bands from the CD70+ membrane preps (lanes 3, 4, 7, and 8). No binding was observed in extracts from parental 293 cells as expected (lane 2 and 6). CD70 is predicted to be trimeric based on its structural homology to TNF-*α*. The three distinct bands detected in membrane extracts of CD70 transfected cells (lanes 3 and 7) are identical to the endogenous CD70 expressed by 786-0 cells (lanes 4 and 8). The molecular sizes of 29 kDa, 55–60 kDa, and >95 kDa are remarkably similar to the pattern described by Lens *et al* (1999) when 2 other anti-CD70 mAbs were used for immunoprecipitation studies. We used *β*-actin as loading control for the cell lysates (Figure 1A, lower panel).

### IHC analysis on normal tissue microarrays

To further test the specificity of the two mAbs for binding to CD70, we carried out IHC analysis on a FFPE tissue microarray of normal human tissues containing at least three different individual cores for each tissue type. As shown in Figure 1B, positive staining was restricted to lymphoid tissues (tonsil, thymus, spleen), and gut-associated lymphoid cells (data not shown) in which activated CD70+ lymphocytes can be found. Normal vital organs such as kidney, pancreas, and liver are negative (Figure 1B). The restricted binding of mAb 1C1 and mAb 5D12 in normal tissues microarrays and western blot analysis contrasted with testing of commercially available CD70 antibodies which gave non-specific binding in CD70-negative cells and tissues (data not shown).

### FACS analysis

**Figure 2:**
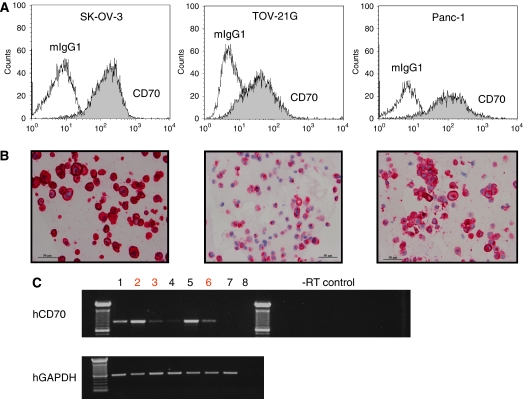
Correlation of CD70 expression in cell lines by qFACS, IHC, and reverse trancriptase–PCR: (**A**) Quantitative flow cytometric analyses were carried out on ovarian (SK-OV-3, TOV-21G), and pancreatic (Panc-1) cell lines using a PE-conjugate murine anti-CD70 ab (BD Pharmingen). CD70 copy number was determined using standard labelled bead controls (QiFiKit, DAKO). (**B**) Corresponding cell pellets were harvested and processed as FFPE samples and immunohistochemistry using 1C1 (as shown) or 5D12 was carried out using alkaline phosphatase-labelled secondary antibody and Fast Red as the chromogen. Images were taken using a Zeiss microscope at 400 × magnification. Scale bar=50 *μ*m. (**C**) Detection of human CD70 expression by reverse trancriptase–PCR: expression of CD70 in various CD70+ cell lines was determined using cDNA preparations obtained from the cells (lanes 1: AN3CA ovarian; 2: SKOV-3 ovarian; 3: TOV-21G ovarian; 4: NCI-H716 colorectal; 5: 786-O renal; 6: PANC-1 pancreatic; 7: IGROV-1 ovarian (CD70 negative); 8: H_2_O). Primers that partially spanned the coding regions were used for CD70. PCR products were sequenced to confirm their identity. hCD70 (*TNFSF7*) fragment=852 bp, including exons 1,2, and 3. Human GAPDH (*hGAPDH)* (580 bp) was used as housekeeping gene PCR control.

To confirm the IHC data, we performed quantitative fluorescence activated cell sorting (qFACS) on various pancreatic and ovarian cancer cell lines using an anti-CD70 mAb that is different from 1C1 and 5D12. The qFACS data provide approximation of CD70 cell surface expression, wheraes IHC will capture both membrane and intracellular CD70 protein expression. Results are shown in Table 1 and Figure 2. *In vitro*, the ovarian cancer cell line SK-OV-3 had the highest level of CD70 detected by qFACS (2.2 × 10^5^ antigens per cell), whereas the Panc-1 cell line had 1 × 10^4^ antigens per cell. Reverse trancriptase–PCR analysis for CD70 expression also showed that SK-OV-3 cells have relatively higher level of CD70 mRNA compared with Panc-1 and TOV-21G cells (Figure 2C). There was a good correlation for detection of CD70 expression levels using three assays (reverse trancriptase–PCR, flow cytometry, and IHC). With the exception of the Panc-1 cell line, four out of the five pancreatic cell lines analysed were negative for CD70 transcript and protein expression.

### CD70 expression in carcinomas

We proceeded to use 1C1 and 5D12 mAbs as IHC reagents to expand our analysis of CD70 expression in various tumour types. We have previously reported finding CD70 expression in about 70% of renal cell carcinoma cases (Law *et al*, 2006). More recently, we also reported on expression of CD70 in other haematologic tumour types (McEarchern *et al*, 2008). Table 2 summarises the results from our analysis of various TMAs. Notably, we observed novel aberrant expression of CD70 in multiple solid tumour types, such as pancreatic (35 out of 140), melanoma (15 out of 96), ovarian carcinomas (37 out of 241), and lung adenocarcinomas (17 out of 172). Most of the melanoma cases have low intensity (1–2+) and a low percentage (1–50%) of CD70+ tumour cells. Our data also confirms high expression of CD70 (70%) in most but not all of renal cell carcinoma cases, consistent with our earlier study using another anti-CD70 mAb (1F6) in frozen tissue microarrays (Law *et al*, 2006). We did observe a lower percentage of CD70-positive cases in nasopharyngeal and brain cancers compared with previously published studies. Our analysis showed 18 out of 82 cases of laryngeal and pharyngeal cancers are positive for CD70 (Table 2), whereas Agathanggelou *et al* (1995) reported that 16 out of 20 nasopharyngeal cancers were CD70 positive. We also found CD70-positive staining in only 6 out of 59 cases of brain cancers. In contrast, Wischhusen *et al* (2002) found that 5 out of 12 glioblastomas and 3 out of 4 anaplastic astrocytomas were positive for CD70 protein.

**Figure 3:**
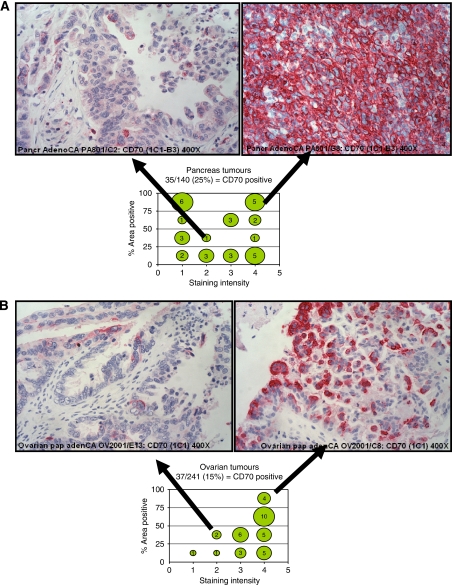
Immunohistochemistry analysis using anti-CD70 mAb (1C1) in tumour microarrays. CD70 expression in pancreatic (**A**) and ovarian (**B**) tumours is shown. Of the 140 and 241 individual cases of pancreatic and ovarian carcinoma, respectively, 35 cases (25%) and 37 cases (15%) were found to be CD70+. In this analysis, the area of transformed cells within each biopsy stained positively for CD70 expression is categorised into <25%, 25–50%, 50–75%, and >75%. The intensity of CD70 expression is expressed as: 1=weak, 2=mild, 3=moderate, 4=strong ( × 400 magnification). To illustrate the heterogeneity in CD70 expression, the percent area positive is plotted against staining intensity. In these plots, the number in each in the graphs denotes the number of CD70+ cases. Representative images for low, heterogeneous (25–50% positive, 2+) and high, homogeneous (>75% positive, 4+) CD70 for pancreatic and ovarian carcinoma are shown.

As shown in Figure 3, the intensity and pattern of CD70 expression in these tumours are highly variable. To describe the variability of expression, we plotted the intensity of CD70 staining against the percentage of tumour cells that stained positive within the biopsy core (Figure 3A and B). For both pancreatic and ovarian tumours, about 14% of all cases showed 3–4+ staining intensity. In contrast, just 1.6% of ovarian cases showed a lower staining intensity of 1–2+, whereas 11% of all pancreatic cases had an intensity of 1–2+ (Figure 3B). CD70+ renal cell carcinomas tumours typically also have homogeneous high-intensity staining (3–4+, data not shown). CD70 was not detected in the normal pancreas and ovary samples tested. Other tumour types that expressed CD70 (Table 2) also showed heterogeneous staining pattern with lower intensity. In a number of cases, we also observed infiltrating leukocytes within the tumours that were CD70+ (data not shown).

### *In vitro* activity of SGN-75 against CD70+ ovarian and pancreatic cell lines

**Figure 4:**
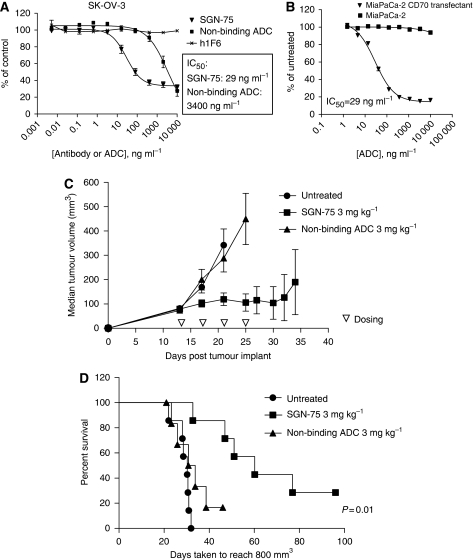
*In vitro* and *In vivo* Anti-tumour Activity of SGN-75: (**A** and **B**) *In vitro* cytotoxicity assay showing potency of SGN-75 (triangles) on SK-OV-3 ovarian and CD70-transfected MiaPaCa-2 pancreatic carcinoma cell lines. The controls, unconjugated h1F6 control (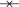) and non-binding ADC (**A**, squares) have no effect. IC_50_ for SGN-75 and non-binding ADC are noted. Similarly, no cytotoxicity is observed in the untransfected MiaPaCa-2 control (**B**, squares). (**C** and **D**) *In vivo* anti-tumour activity of SGN-75 in0-transfected MiaPaCa-2 pancreatic carcinoma tumours in nude mice. Mice treated with SGN-75 (squares) showed a significantly reduced median tumour volume (**C**) and a higher percent survival (**D**) when compared with untreated (circles) and non-binding ADC (triangles).

We have previously reported that mAb-drug conjugates (ADC) targeting CD70 on the cell surface deliver a highly potent cytotoxic antitubulin agent, monomethyl auristatin F (MMAF). This anti-CD70 ADC, designated as SGN-75, is a humanised mAb (h1F6) that has an average of four drugs (MMAF) attached by a maleimidocaproyl (mc) linker (h1F6-mcMMAF(4)) (Oflazoglu *et al*, 2008). On internalisation of the ADC, the drug cys-mcMMAF is released (Alley *et al*, 2009), resulting in disrupted tubulin polymerisation and apoptosis. To determine whether SGN-75 can inhibit the growth of ovarian and pancreatic cancer cells, we carried out *in vitro* cytoxicity assays using ovarian cancer cell lines SK-OV-3 and TOV-21G and a pancreatic cancer cell line Panc-1. In the ovarian cell line SK-OV-3, SGN-75 induced dose-dependent cytotoxicity that is specific with an IC_50_=29 ng ml^−1^ (Figure 4A). Unconjugated 1F6 had no effect, whereas the non-binding ADC control showed some cell killing but only at the highest doses tested (>1000 ng ml^−1^, IC_50_=3400 ng ml^−1^), suggesting that specific cell killing was mediated by SGN-75 subsequent to its binding to CD70.

SGN-75 had no significant impact on the viability of TOV-21G cells or the pancreatic cell line Panc-1 (data not shown) possibly because of the relatively low level of CD70 expression found on these cells when compared with SK-OV-3 cells (Table 1). Hence, validating the anti-tumour activity of SGN-75 on pancreatic cell lines presented a challenge because within the panel of pancreatic cell lines studied, only Panc-1 cells express CD70 (Table 1). To overcome this obstacle, we engineered another pancreatic cell line MiaPaCa-2 to express CD70 (designated as MiaPaCa-2/CD70). MiaPaCa-2/CD70 is a stable cell line that expressed high levels of CD70 as determined by qFACs (Table 1). MiaPaCa-2/CD70 cells were effectively killed by SGN-75 *in vitro* with an IC_50_=29 ng ml^−1^, whereas the parental cell line that lacked CD70 was unaffected (Figure 4B). Taken together, these *in vitro* studies showed that SGN-75 has the potential to kill ovarian and pancreatic cancer cell lines that express moderate to high levels of CD70.

### *In vivo* activity of SGN-75 against MiaPaCa-2/CD70

Next, we tested the anti-tumour activity of SGN-75 *in vivo* using CD70+ tumour xenografts. Model development with SK-OV-3 revealed that expression of CD70 was significantly down modulated in subcutaneous tumour xenografts with a lack of consistency between tumour samples (data not shown). For this reason, we used MiaPaCa-2/CD70 which retained high expression of CD70 *in vivo*. Mice were dosed with either 3 mg kg^−1^ of SGN-75 or a non-binding control ADC every 4 days for a total of four doses starting when tumour volumes reached 100 mm^3^. Administration of SGN-75 significantly reduced median tumour volumes and delayed tumour growth when compared with untreated controls or a non-binding ADC (Figure 4C). SGN-75 treated mice also showed statistically significant prolonged survival (median survival of 60 days) compared with either untreated control group (30 days) or non-binding ADC (32 days) (*P*=0.01) (Figure 4D), thus confirming the *in vivo* anti-tumour activity of SGN-75. Notably, there were two out of seven mice, in which the tumours completely regressed with SGN-75 treatment.

## Discussion

CD70 is a rapidly internalising cell surface antigen that shows a minimal expression on normal tissue but is frequently upregulated on haematologic malignancies (Al Saati *et al*, 1989; Lens *et al*, 1999; McEarchern *et al*, 2007), nasopharygeal carcinomas (Agathanggelou *et al*, 1995), and renal cell carcinomas (Junker *et al*, 2005; Diegmann *et al*, 2006; Law *et al*, 2006). These qualities make CD70 an attractive therapeutic target for an anti-CD70 ADC. Although CD70 expression is prevalent in many tumour types, the percentage of CD70-positive cases varies. Thus, protein expression must be confirmed in individual tumour samples. This has been a significant obstacle for analysis of CD70 in pathologic specimens because most archival tissues are preserved as FFPE samples, in which existing anti-CD70 antibodies fail to work. Earlier studies on the expression of CD70 in cancers have relied on flow cytometric analysis for haematologic malignancies and IHC on limited frozen tissues for carcinomas. Our published study on renal cell carcinomas used the murine and humanised form of our therapeutic anti-CD70 (1F6 mAb) as primary antibody for detection of CD70 expression in frozen tissues and, up to this point, represented the most extensive analysis of CD70 protein expression in renal cell carcinoma. But 1F6, similar to the commercially available antibodies that we have tested, only gave specific binding to frozen tissues and not FFPE samples. Similarly, our attempts to prepare polyclonal antibodies against distinct peptides spanning most of the extracellular and intracellular domains of CD70 also proved to be disappointing because they tended to yield FFPE-reactive antibodies that lacked specificity for CD70.

We overcame the obstacle of profiling CD70 in archived specimens by generating anti-CD70 antibodies that are suitable for detection of CD70 in FFPE tissues. Our successful approach incorporated immunisation of denatured antigen with a novel, high throughput screening strategy designed to identify antibodies that specifically bound to formalin-denatured CD70. The specificity of these antibodies was tested using western blot analysis and comparative binding to known CD70-positive and -negative cell lines as well as normal tissues. The antibodies 1C1 and 5D12 consistently showed sensitive and specific binding to CD70 in FFPE samples of CD70-positive normal human lymphoid tissues and tumours. In agreement with previous observations, 1C1 and 5D12 showed that expression of CD70 is limited to lymphoid organs, in which activated T and B cells are found. Using these reagents we were able to extensively analyse CD70 expression in multiple tumour types. In addition, our expanded IHC analysis of CD70 identified other indications, in which an anti-CD70 ADC could potentially be tested, most notably a significant fraction of pancreatic and ovarian carcinoma cases. In agreement with our ovarian carcinoma data (Figure 3B and Table 2), CD70 has recently been reported as a potential cisplatin resistance marker in ovarian cancer using proteomic methods (Aggarwal *et al*, 2009).

We did observe discrepancy with the published literature on the frequency of CD70 expression in nasopharyngeal and brain cancers. Our analysis showed that 18 out of 82 cases of laryngeal and pharyngeal cancers are positive for CD70 compared with 16 out of 20 observed by Agathanggelou *et al* (1995). Their study noted that 20 out of 20 of the nasopharyngeal cancers were positive for Epstein–Barr virus (EBV). We did not examine the EBV status of the laryngeal and pharyngeal cancers in our TMAs. However, EBV transformation causes up-regulation of CD70 in B cells (Herbst *et al*, 1996) and may contribute to the higher percentage of CD70+ nasopharyngeal cancers observed by Agathanggelou *et al* (1995). We also found that only 6 out of 59 (10%) of brain cancer cases were CD70+, whereas Wischhusen *et al* (2002) found that 5 out of 12 of glioblastomas and 3 out of 4 anaplastic astrocytomas were positive for CD70 protein. The limited number of cases examined by Wischhusen *et al* may contribute to this discordance of the data.

Our studies extend beyond a survey of CD70 in solid tumour specimens by validating the anti-tumour activity of SGN-75 in these indications. In preclinical studies, we have demonstrated *in vitro* anti-tumour activity of the anti-CD70 antibody-drug conjugate SGN-75 in CD70+ ovarian and pancreatic cell lines. Anti-tumour activity of SGN-75 correlates with higher levels of CD70 expression, suggesting that expression may be one factor that contributes to its activity in solid tumour indications. Although the percentage of CD70+ in pancreatic cell lines (20%) is close to what we observed in primary pancreatic tumours (25%), the low level CD70 expression in a pancreatic cell line (Panc-1: 10 000 CD70 receptors per cell) is not fully consistent with the expression we observed in primary pancreatic tumours (Table 2 and Figure 3A) and it may reflect the inability of monolayer cultures to faithfully reproduce the expression pattern and architecture of the primary tumour (Sipos *et al*, 2004; Froeling *et al*, 2009). Carcinoma cell lines expressing ⩽10 000 copies of CD70 per cell did not show *in vitro* activity of SGN-75. In contrast, SK-OV-3 and MiaPaCa-2/CD70 cells that have high levels of CD70 expression (Table 1), showed a potent IC_50_ of 29 ng ml^−1^, which corresponds to approximately 0.77 nM delivered drug. The anti-tumour activity of SGN-75 was highly specific and occurred at a concentration that is well below the level required to reach saturation binding *in vitro*. Furthermore, SGN-75 showed statistically significant anti-tumour activity (*P*=0.01) in mouse xenograft models of pancreatic carcinoma when compared with the non-binding control-treated mice. All seven mice treated with SGN-75 showed a delay in tumour growth, whereas two of seven mice showed a complete and sustained regression. This is the first reported study of anti-CD70 ADC anti-tumour activity in pancreatic cancer.

The targeting of CD70 by SGN-75 is thought to be driven by the delivery of cys-mcMMAF to the tumour. However, h1F6, the antibody backbone for SGN-75, also mediates complement-dependent cytotoxicity and engages effector cells by Fc*γ* receptor to induce tumour cell lysis (McEarchern *et al*, 2007). The effector-function-based activities of h1F6 are retained in the ADC (J McEarchern and C Law, unpublished observations), suggesting that SGN-75 has the potential to mediate anti-tumour activity in CD70+ tumours through multiple mechanisms.

In earlier reports, we have showed SGN-75 anti-tumour *in vivo* activity in models of renal cell carcinoma and NHL. The high percentage of renal cell carcinomas and NHL samples that express CD70 provides a strong rationale for clinical testing and a phase I clinical trial of SGN-75 in these patient populations is currently in progress. The results of this study support extending the clinical study of SGN-75 to pancreatic and ovarian cancer patients with tumours that express CD70. However, clinical testing of SGN-75 in these malignancies must be coupled with an effective method to prospectively evaluate the effect of variable CD70 expression on anti-tumour activity. We are currently looking at CD70 tumour expression on patients entering our Phase I clinical trial. It may be possible to identify a threshold of CD70 intensity or uniformity of expression that will allow for improved patient selection. We believe that the results reported in this study provide a rationale for testing CD70-directed drug delivery in new indications and provide novel reagents for the development of screening tests.

## Figures and Tables

**Table 1:** Comparative CD70 expression in pancreatic and ovarian cancer cell lines by immunohistochemistry and quantitative FACS

**Cell line**	**Tumor type**	**CD70 IHC (1C1)**	**CD70 IHC (5D12)**	**CD70 receptor number**^a^ **( × 10^3^)**
OVCAR-3	Ovarian	1+	1–2+	1.3
SK-OV-3	Ovarian	3+	3+	223
CaOV3	Ovarian	1+	2+	0.3
TOV-21G	Ovarian	1+	1+	2
CAPAN-1	Pancreatic	Negative	Negative	0
CAPAN-2	Pancreatic	Negative	Negative	0
SU86.86	Pancreatic	Negative	Negative	0
Panc-1	Pancreatic	2+	2+	10
HPAF-II	Pancreatic	Negative	Negative	0
MiaPaCa-2/CD70	Pancreatic CD70 transfectant	3+	ND^b^	366

Abbreviations: IHC=immunohistochemistry; ND=not determined.

a Determined by quantitative FACs.

b No data.

**Table 2:** Summary of CD70 expression from tissue microarray analysis of carcinomas using 1C1

**Tumor type**	**CD70+ of total**	**%CD70+**
Kidney^a^	204 of 283	72
Pancreas	35 of 140	25
Larynx or pharynx^b^	18 of 82	22
Melanoma	15 of 96	16
Ovary	37 of 241	15
Lung adenocarcinoma	17 of 172	10
Colon	17 of 194	9
Breast	5 of 204	2
Brain	6 of 59	10

a 189 of 230 (82%) clear cell carcinoma and 4 of 8 (50%) papillary carcinoma.

b Includes nasopharyngeal cancer.
